# Stereotest Comparison: Efficacy, Reliability, and Variability of a New Glasses-Free Stereotest

**DOI:** 10.1167/tvst.9.9.29

**Published:** 2020-08-20

**Authors:** Alice Grasso McCaslin, Kathleen Vancleef, Luke Hubert, Jenny C A. Read, Nicholas Port

**Affiliations:** 1 School of Optometry, Indiana University, Bloomington, IN, USA; 2 Institute of Neuroscience, Newcastle University, Newcastle Upon Tyne, UK

**Keywords:** stereoacuity, stereopsis, binocular vision, vision tests, depth perception

## Abstract

**Purpose:**

To test the validity of the ASTEROID stereotest as a clinical test of depth perception by comparing it to clinical and research standard tests.

**Methods:**

Thirty-nine subjects completed four stereotests twice: the ASTEROID test on an autostereo 3D tablet, a research standard on a VPixx PROPixx 3D projector, Randot Circles, and Randot Preschool. Within 14 days, subjects completed each test for a third time.

**Results:**

ASTEROID stereo thresholds correlated well with research standard thresholds (*r* = 0.87, *P* < 0.001), although ASTEROID underestimated standard threshold (mean difference = 11 arcsec). ASTEROID results correlated less strongly with Randot Circles (*r* = 0.54, *P* < 0.001) and Randot Preschool (*r* = 0.64, *P* < 0.001), due to the greater measurement range of ASTEROID (1–1000 arcsec) compared to Randot Circles or Randot Preschool. Stereo threshold variability was low for all three clinical stereotests (Bland–Altman 95% limits of agreement between test and retest: ASTEROID, ±0.37; Randot Circles, ±0.24; Randot Preschool, ±0.23). ASTEROID captured the largest range of stereo in a normal population with test–retest reliability comparable to research standards (immediate *r* = 0.86 for ASTEROID vs. 0.90 for PROPixx; follow-up *r* = 0.68 for ASTEROID vs. 0.88 for PROPixx).

**Conclusions:**

Compared to clinical and research standards for assessing depth perception, ASTEROID is highly accurate, has good test–retest reliability, and measures a wider range of stereo threshold.

**Translational Relevance:**

The ASTEROID stereotest is a better clinical tool for determining baseline stereopsis and tracking changes during treatment for amblyopia and strabismus compared to current clinical tests.

## Introduction

Binocular vision has been shown to be superior to monocular vision when performing complex visual tasks.^1^^–^^4^ The primary advantage of binocular vision lies in the neural combination of the visual overlap between the two eyes and the resulting perception of depth. Because a human's two eyes are placed in different horizontal positions on the head, each eye perceives the same three-dimensional (3D) scene from a slightly different angle. The brain measures this horizontal disparity between the images of the two eyes and processes these images to produce a sensation of depth.^5^ The term “stereopsis” refers to the impression of depth arising from binocular disparity and represents one of the truly binocular cues to depth.^6^ Stereopsis can be separated into two distinct forms: global and local.^7^ Local stereopsis can be probed with simple targets containing contour elements. The contour targets in such stereograms contain some monocular cues, allowing them to be solved by patients with abnormal binocular vision.^8^ Global stereopsis, on the other hand, must be probed with more complex targets that lack monocular cues.

The standard quantitative measure of stereopsis is the stereo threshold, or stereoacuity. This is the smallest depth interval between two stimuli that a subject can detect using only stereoscopic cues.^9^ It is generally considered best to measure stereopsis using stimuli in which binocular disparity is the only cue to depth, such as random-dot stereograms (RDSs). In an RDS, the image structure is defined solely by the correlation between left and right eye images; the random dot textures of the stereograms contain no object or contour structures when viewed monocularly.^7^^,^^10^^,^^11^ Random-dot stereograms have an advantage over other tests of stereo threshold that are used in clinical or research settings, as they provide no monocular cues to depth^8^^,^^12^ and therefore probe global stereopsis.

A psychophysical, research standard stereoacuity test would be the most effective way to measure stereo threshold. Such a test would require specialized equipment (such as a large, bulky 3D projector) to isolate the binocular vision system, a dedicated laboratory set-up, trained personnel, and a long and intensive test procedure to ensure the best measurement.^13^ Shorter tests that are easier to administer are more useful for clinical practice, as they can be used as quick screening tests for binocular vision problems. These shorter clinical tests (e.g., Randot Circles, Randot Preschool) are presented as books, which are certainly less expensive and not nearly as bulky as a large projector system. These tests have their own drawbacks,^14^ as they can contain artifacts^15^ such as monocular cues,^16^ only classify stereo threshold into a set number of discrete values, and have poor test–retest reliability.^17^^–^^19^

To solve the known limitations of Randot Circles and Randot Preschool stereotests (Stereo Optical Company, Inc., Chicago, IL) and to create a more entertaining and adaptive test, a group of researchers at Newcastle University, UK, has developed a new stereotest, ASTEROID (short for Accurate STEReotest On a mobIle Device), in the form of a game on a glasses-free 3D tablet.^20^ This colorful, interactive test uses an adaptive Bayesian staircase starting at 1000 arcsec to determine each patient's stereo threshold at a fine sampling resolution.

The aim of this study was, primarily, to test the validity of the ASTEROID stereotest as a viable clinical test of depth perception. We approached this in the following ways: first, we compared the ASTEROID stereotest (taken on a 3D tablet) to our research standard test: a psychophysical stereotest presented on a state-of-the-art 3D projector. Then, we compared the ASTEROID stereotest to the current clinical standards, Randot Circles and Randot Preschool. Finally, we analyzed and quantified test–retest reliability to determine the variability of the ASTEROID, Randot Circles, and Randot Preschool stereotests.

## Methods

Thirty-nine subjects were recruited from family and friends of the authors with Indiana University (IU) School of Optometry affiliation, faculty and students of the IU School of Optometry, and records from past studies at the Borish Center for Optometric Research at the IU School of Optometry. Of these subjects, 30 were classified as “stereo-normal,” with ages ranging from 5 to 46 years and no reported problems with depth perception. The remaining nine subjects were classified as “stereo-abnormal,” and their ages ranged from 7 to 47 years. Their stereo-abnormal status was either self-reported (in the five subjects recruited from IU School of Optometry faculty, staff, and students) or proven from past studies (in the four subjects recruited from Borish Center records). All subjects had no reported visual or ocular problems aside from stereopsis, and any subjects with refractive error or presbyopia wore their habitual correction (either glasses or contact lenses). All subjects gave informed written consent (minors had parental written consent) to participate in the study after receiving an explanation of the nature and possible consequences. This study followed the tenets of the Declaration of Helsinki and was approved by the Institutional Review Board of IU.

For this study, each subject was scheduled for two visits to our laboratory, separated by 5 to 14 days. The stereoacuity of each subject was measured by the same examiner (either AGM or LH) at both visits. The second visit was scheduled for within 14 days to allow for ease of fitting visits into busy schedules, and for 35 of the 39 subjects we were able to achieve this. Two subjects (both stereo-normal children) returned for their second visit more than 14 days after their first visit due to scheduling difficulties. One of these subjects returned 21 days after the first visit; the other returned 42 days after the first visit. Two additional subjects (one stereo-normal child, one stereo-abnormal) were lost to follow-up and did not return for their second visit. For these two subjects, we used the data from their first visit for data analysis.

### Stereotest Comparison

For each test cycle, the subject completed four different stereotests: the ASTEROID tablet-based stereotest, a similar computerized stereotest on a VPixx PROPixx 3D projector (VPixx Technologies, Saint-Bruno, Quebec, Canada), the Randot Circles stereotest, and the Randot Preschool stereotest. The 3D projector test (PROPixx) threshold was considered the subject's true stereo threshold, as it represents a standardized psychophysical stereotest. The ASTEROID, Randot Circles, and Randot Preschool thresholds were compared to the PROPixx threshold to determine their accuracy. The order in which these stereotests were taken was randomized, except that in all cases, the Randot Circles and Randot Preschool tests were administered back-to-back due to required lighting conditions. The protocols for each test are described in detail below.

#### ASTEROID and PROPixx Stereotests

To ensure that the tablet-based and projector-based tasks were as similar as possible, the ASTEROID stereotest code (written in Unity; http://unity.com; compiled to an Android APK) was adapted for MATLAB (MathWorks, Natick, MA) to enable its use on a computer and projector. This allowed both stereotests to perform the same way, using the same staircase format, to determine stereo threshold.

**Image 1.**
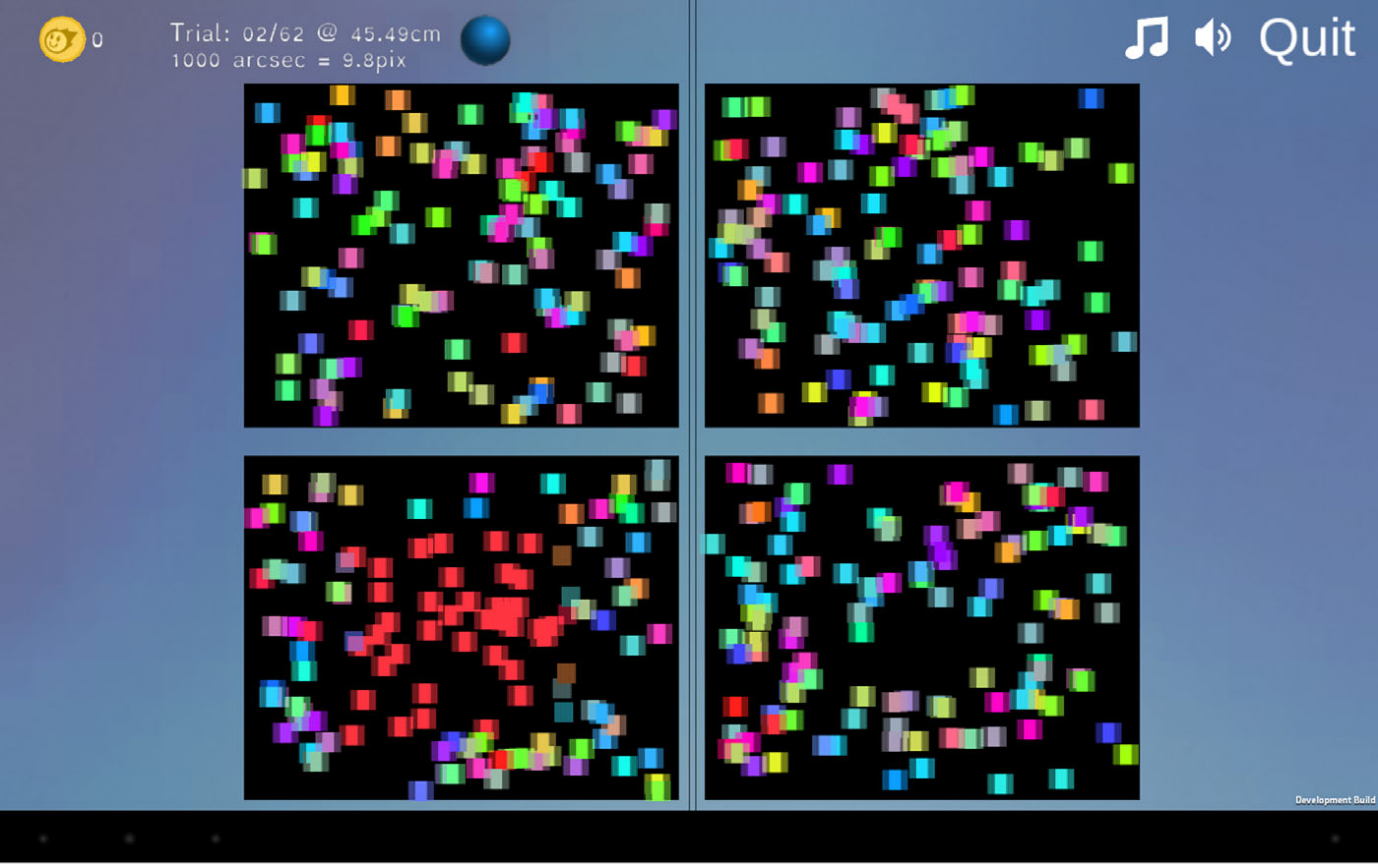
ASTEROID tablet screen. Screenshot from ASTEROID version 0.931 during the second (1000 arcsec) trial with a monocular cue. The bottom left panel shows a *red colored square*, which would appear as if it were coming out toward the observer if viewed on a Commander 3D tablet.

Both the ASTEROID tablet test and the 3D projector test consisted of 65 four-alternative forced-choice trials. In each trial, four panels of dynamic random dots with a refresh rate of 60 Hz were presented to the subject on the screen; one of those four panels had a square apparently floating in depth in front of the screen at a given disparity (Image 1; see Ref. 20 for a full description). The subject determined which panel the floating square was in and indicated the panel he or she chose. All trials were self-paced with no time limit. After the subject indicated his or her choice, the test moved on to the next trial. If the subject chose the correct panel containing the floating square, the next trial would present a square at a smaller disparity. If the subject chose incorrectly, the next trial would present a square at a larger disparity. Initially, the subject was shown five practice trials with a monocular cue (a colored square) shown at various depths in a fixed-step staircase, starting at 1000 arcsec and decreasing disparity in logarithmic steps by factors of 1.4.^20^ After these practice trials were completed, the test continued for 60 trials in an adaptive staircase without the monocular color cue, utilizing a finer step size. The ASTEROID stereo threshold, which can be any value between 1 and 1200 arcsec,^20^ was determined at the end of a 60-trial adaptive Bayesian staircase, which has been proven to be an efficient method to determine sensory thresholds.^22^

#### ASTEROID Stereotest

The ASTEROID tablet stereotest (version 0.931, using the long 60-trial option) was administered on a Commander 3D glasses-free tablet computer (Toronto, ON, Canada),^23^ which uses parallax-barrier autostereoscopic 3D to present images in depth on a 10.1-inch screen.^20^ The subject kept his or her chin in a chinrest, and the tablet was set on a stand 45 cm from the subject's chin; this was done to keep the test distance standardized and stable among all subjects and to replicate as closely as possible the protocol with the 3D projector, although the ASTEROID stereotest does not require a stand nor a chinrest for use. This testing distance resulted in the following visual angle specifications (horizontal × vertical, assuming subject fixation on the center of the test area): 8.53° × 6.73° per panel, 17.72° × 14.22° for the entire test area, 4.28° square for the 3D box, and 0.446° square for the colored dots. The tablet stand was weighted to prevent movement and was positioned parallel to the subject's face; this ensured that differences in parallax between the two eyes did not interfere with the subject's ability to perceive depth and that the subject was correctly positioned with respect to the tablet's parallax barrier, so that each eye was seeing the image intended for it. The subject wore a sticker bearing a high-contrast geometric design on the center of his or her forehead, roughly equidistant between his or her eyes, to allow the tablet to track the distance (based on the apparent size of the sticker in the front-facing camera image)^20^ from the subject's eyes. To reduce glare off of the tablet, the overhead room lights were turned off during the ASTEROID testing procedure. One small light was kept on elsewhere in the testing room to allow the examiner to move freely about the room in the dark; otherwise, all light came from the tablet (average luminance 8.75 cd/m^2^). In the ASTEROID test, the subject was asked to tap the panel on the screen of the Commander 3D tablet where he or she saw the floating square. Auditory and visual feedback was given after each choice; a correct choice was acknowledged by confetti, celebratory sounds (e.g., applause), and an animation of a box opening to reveal a prize. An incorrect choice was indicated by a low-pitched tone, and a box appeared in the correct location but remained closed.

#### PROPixx Stereotest

The appearance of the 3D projector stereotest was very similar to that of the tablet test, although it was presented on a larger screen (image size 83.5 cm width by 47 cm height) at a distance farther from the subject's face. To keep visual angles similar between the tablet test and the projector test, the subject placed his or her chin in a chinrest located 140 cm from the projector screen. This testing distance resulted in the following visual angle specifications (horizontal × vertical, assuming subject fixation on the center of the test area): 8.86° × 6.66° per panel, 18.74° × 14.13° for the entire test area, 4.21° square for the disparate target, and 0.273° round for the colored dots. The projector screen was positioned parallel to the subject's face plane to ensure that differences in parallax between the two eyes did not interfere with perception of depth. The test was projected from a VPixx PROPixx projector, which has a native resolution of 1920 × 1080 and uses circularly polarized light, passive polarized glasses, and alternating right and left eye images (refresh rates up to 500 Hz) to produce a 3D image.^24^ As in the tablet test, the subject had to determine which of four squares contained a target floating in depth in front of the screen. The subject was asked to press a key on a wireless keyboard that corresponded to the location of the floating square (“A” for top left, “Z” for bottom left, “K” for top right, or “M” for bottom right), rather than touching the square as they did for the tablet test. In this projector-based test, only auditory feedback was given for each choice. A high-pitched, 2000-Hz sine-wave tone denoted a correct choice, and a low-pitched, 200-Hz sine-wave tone denoted an incorrect choice.

#### Randot Circles Stereotest

The Randot Circles stereotest was administered as described in the Randot stereotest instruction guide.^25^ The subject held the Randot test book under full room light (luminance 212 * π cd/m^2^) at a distance of 40 cm from his or her eyes while wearing the polarized testing glasses. The subject was shown the multiple-choice series of Wirt circles to test fine depth discrimination and was asked which circle, of the three circles per line, seemed to float forward. Each subject's answers for the Wirt circle section of the Randot test were compared to the scoring key on the back of the test booklet to determine his or her Randot Circles stereo threshold. The subject's Randot Circles stereo threshold was the last correctly identified level on the Wirt circle task; possible scores on the Wirt circle task were 20, 25, 30, 40, 50, 70, 100, 140, 200, or 400 arcsec.

#### Randot Preschool Stereotest

The Randot Preschool stereotest was administered as described in the Randot Preschool stereotest instruction guide.^21^ Each subject held the Randot Preschool test book under full room light at a distance of 40 cm from his or her eyes while wearing the polarized testing glasses. The subject was first shown the first page of the booklet, the 200- and 100-arcsec threshold levels. The subject was asked to identify all of the shapes at the 200-arcsec level; subjects who correctly identified at least two out of the three shapes at that level were asked to repeat the task at the 100-arcsec level. If the subject was again able to identify two out of the three shapes at the 100-arcsec level, the process was repeated with the second page, the 60- and 40-arcsec threshold levels. If the subject was unable to identify two out of the three shapes at the 200-arcsec level, the process was repeated with the third page, the 800- and 400-arcsec threshold levels. The subject's Randot Preschool stereo threshold was the smallest level at which they could identify at least two out of the three shapes; possible scores on the Randot Preschool task were 40, 60, 100, 200, 400, or 800 arcsec.

### Ideal Number of Trials

To determine the best number of trials for the PROPixx test and, consequently, for the similar ASTEROID stereotest, the working stereo threshold (or disparity) for each trial was compared to the subject's “true” stereo threshold determined at the end of the 60 stereo trials (ignoring the initial five practice trials). Doing so measured the loss of accuracy in threshold estimation as the number of trials was shortened. The ideal number of trials was the minimum number necessary to obtain an accurate estimate of the “true” stereo threshold.

### Test–Retest Reliability

At the first visit, after each subject completed all four stereotests, he or she repeated all four a second time in the same order. This was done to determine any potential learning effects that the subject might have experienced after completing the tests once. At the subject's second visit, the subject repeated the four stereotests in the same order as at the first visit. The threshold determined by each stereotest was compared to the first threshold from the same test. This was done to determine the test–retest reliability of each stereotest.

### Statistical Analysis

We performed statistics on log thresholds, rather than threshold values, as log thresholds are closer to normally distributed (see Ref. 26 for further details). Similarly when combining different thresholds obtained for the same subject, we took the mean of log thresholds (equivalent to the geometric mean of the thresholds). We used Pearson's correlation coefficient on log thresholds to compare the test–retest reliability of each stereotest, as well as to compare stereo thresholds among the different stereotests. We also used paired *t*-tests on log thresholds to compare thresholds determined by different stereotests.

Bland–Altman plots were constructed to compare the stereo thresholds across time points (for test–retest reliability) or different stereotests (for stereotest comparison). Bland–Altman analysis is the standard for assessing the level of agreement between two different methods of clinical measurements or to assess the repeatability of one method of clinical measurement.^27^ Agreement between measures is considered good when the mean difference (mean delta) is close to zero, whereas the repeatability of an individual test is determined by the standard deviation (SD) of the difference distribution: 1.96 × SD = coefficient of repeatability.

## Results

### Stereo Thresholds Obtained by Each Test

**Figure 1.**
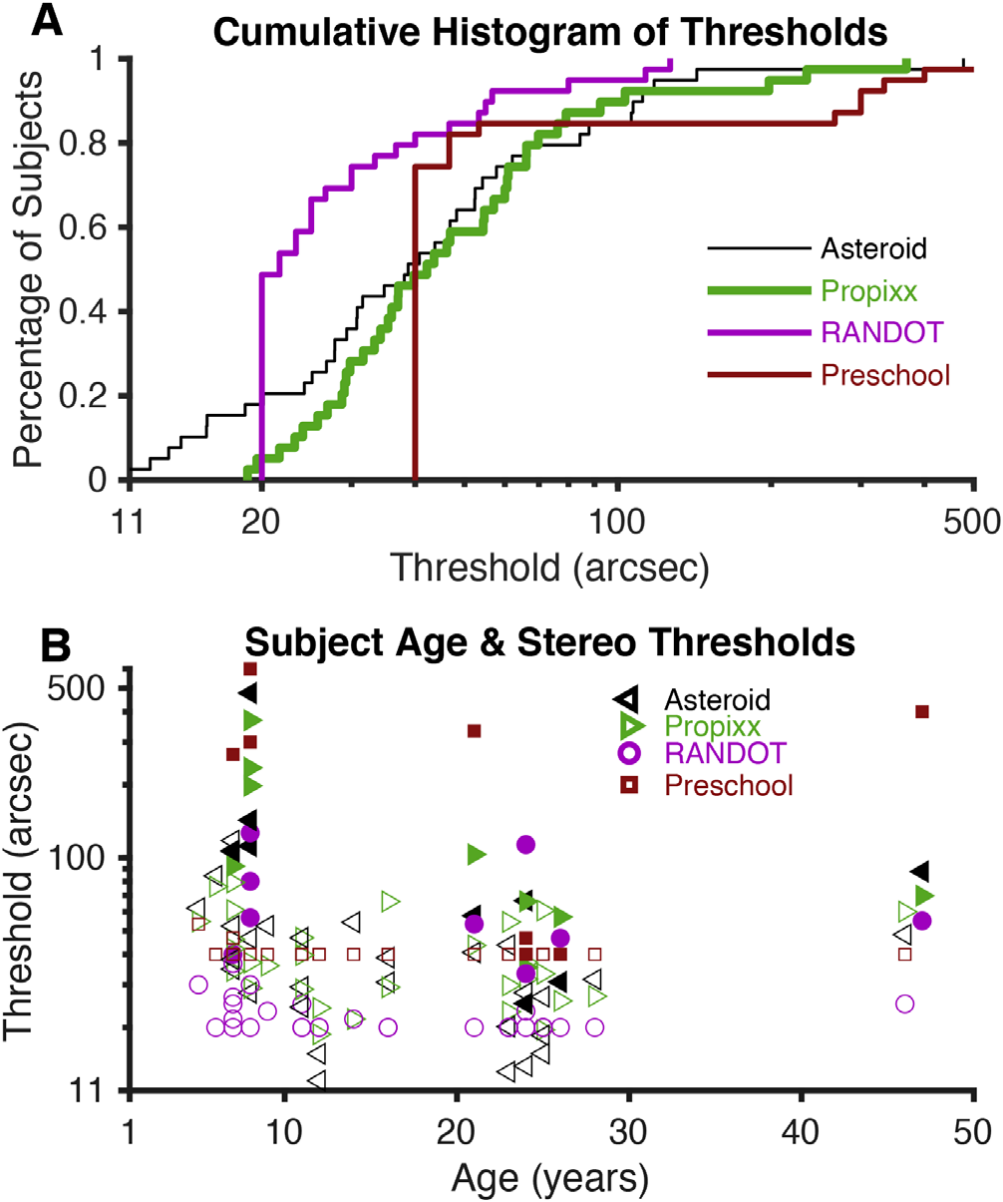
Stereo thresholds. (**A**) Plots of the stereo thresholds for all 39 subjects, as determined by each test (geometric mean of three thresholds for each subject), as cumulative histograms. The two subjects who did not return for follow-up visits are included, but the data were averaged over two measurements instead of three. (**B**) Plots of the stereo thresholds for each subject as a function of his or her age. *Filled shapes* represent stereo-abnormal subjects; *non-filled shapes* represent stereo-normal subjects.

**Figure 2.**
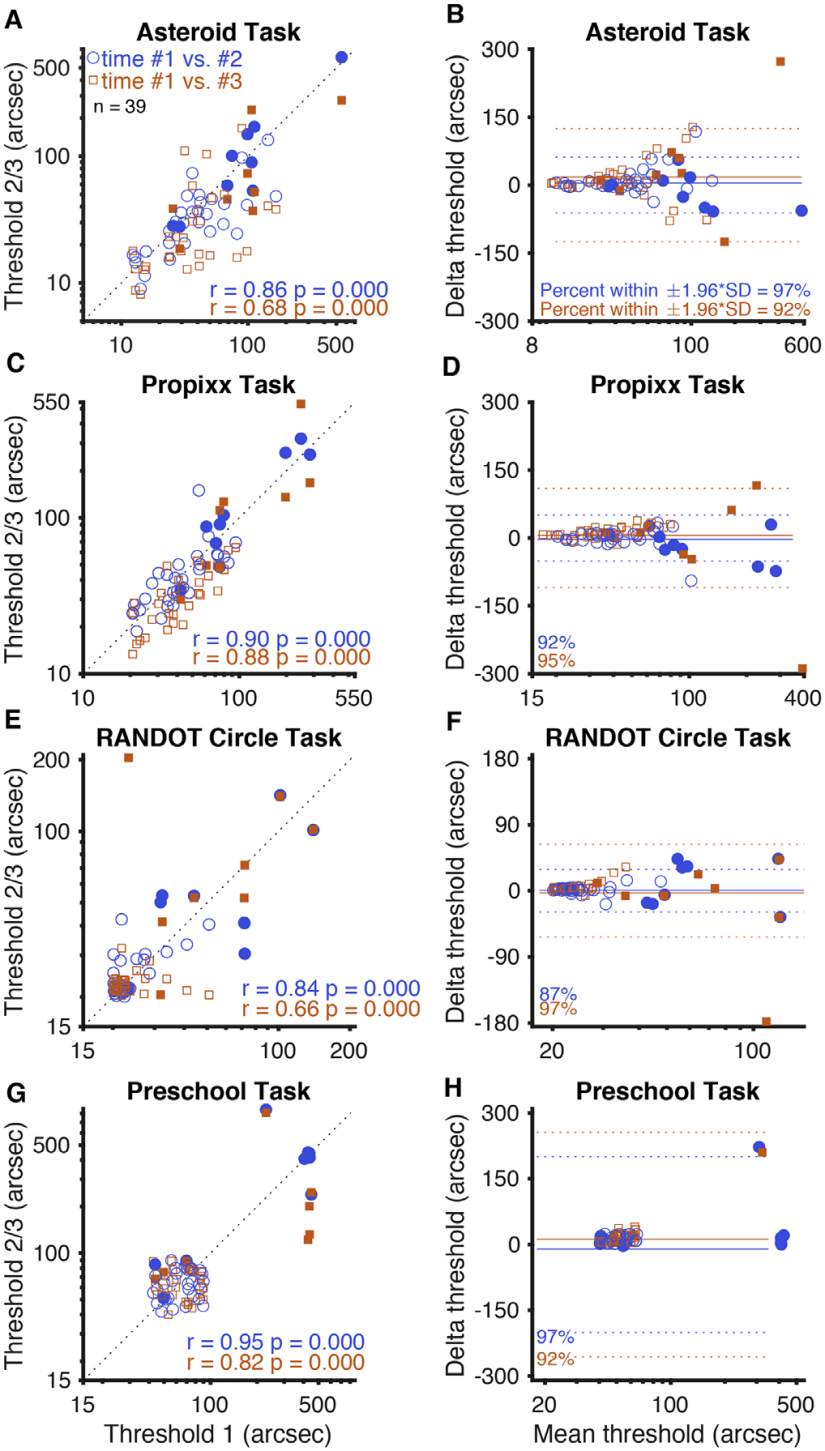
Test–retest reliability. (**A**, **C**, **E**, **G**) Graphs on the *left side* of the figure represent threshold values determined by each test for each subject, plotted with a line representing perfect 1:1 correlation. (**B**, **D**, **F**, **H**) Bland–Altman plots for each test on the *right side* of the figure show mean delta (solid line) and 95% limits of agreement, or ±1.96 × SD (*dotted lines*). *Blue circles* and *lines* represent each subject's second attempt at the test plotted against their first attempt. *Orange squares* and *lines* represent each subject's third attempt at the test plotted against their first attempt (approximately 14 days later). The two subjects who did not return for follow-up visits are included but do not contribute to the *orange squares*. Randot Circles and Randot Preschool points are jittered, but all statistics are computed from the raw data (log_10_ transformed). *Filled shapes* represent stereo-abnormal subjects; *non-filled shapes* represent stereo-normal subjects.

**Figure 3.**
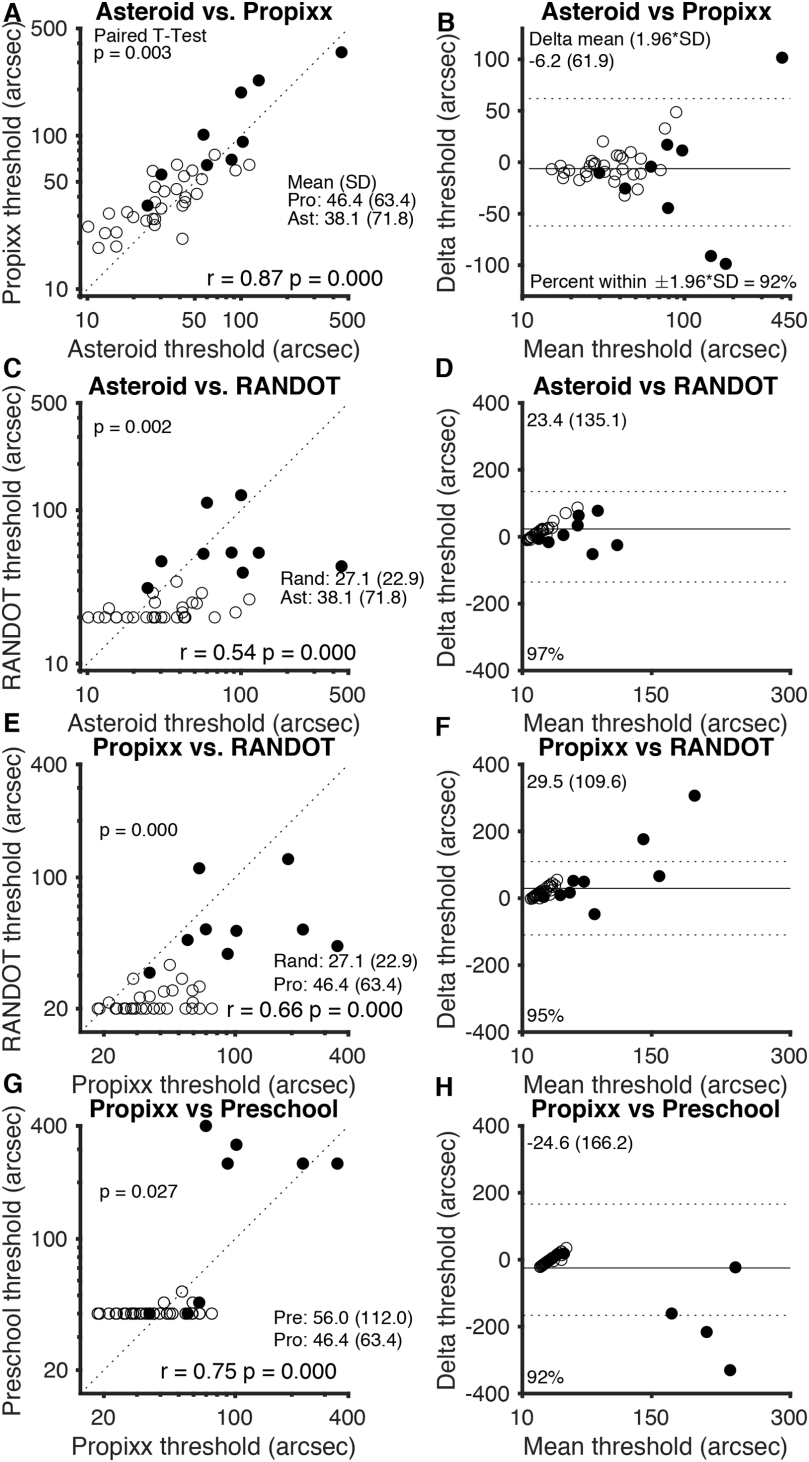
Comparison of stereo tests. (**A**, **E**, **G**) Graphs on the *left side* of the figure plot thresholds of each clinical test (ASTEROID, Randot Circles, and Randot Preschool) against thresholds for the research standard (PROPixx) test. (**C**) Plot of ASTEROID against the commonly used Randot Circles stereotest thresholds; a *dotted line* representing perfect agreement is plotted on each graph. (**B**, **D**, **F**, **H**) Bland–Altman plots on the right side of the figure show mean delta (*solid line*) and 95% limits of agreement (*dotted lines*) for each comparison. The geometric means of the three time points of data collection for each subject were used for this analysis; the geometric means and traditional standard deviations are listed for each stereotest. The two subjects who did not return for follow-up visits are included but data were averaged over two measurements instead of three. *Filled shapes* represent stereo-abnormal subjects; *non-filled shapes* represent stereo-normal subjects.

Figure 1A shows the stereo threshold for our 39 subjects for the four tasks as cumulative histograms, with the three data collection time points for each stereotest in each subject averaged together. For each subject, systematic changes between test sessions were small compared to the measurement error on each session (as seen in Fig. 2). Thus, the average of the three measurements provided a more accurate estimate of each participant's threshold than the first measurement alone. The ASTEROID threshold curve is initially shifted leftward at smaller threshold values, suggesting that subjects with very good stereoacuity obtained even smaller (better) thresholds on ASTEROID than on PROPixx (see also Fig. 3); however, both the PROPixx and the ASTEROID stereotests measure thresholds over 40 arcsec similarly. There is a systematic leftward shift of the Randot Circles curve compared to the other three curves, meaning that the Randot Circles stereotest estimated thresholds to be lower than those measured by the other three stereotests. The Randot Circles stereotest did not accurately reflect each subject's true stereo threshold as determined by PROPixx (Fig. 3). Seventy-two of the 115 Randot Circles thresholds, or 62%, were measured as 20 arcsec, the lowest score possible on the test. The Randot Preschool threshold curve matches the ASTEROID and PROPixx curves more closely, but it exhibits the same saturation as the Randot Circles curve. Ninety-three of 115 Randot Preschool thresholds, or 81%, were measured as 40 arcsec, the lowest score possible on that test.

Figure 1B plots stereo thresholds, as measured by each test, as a function of age. The largest threshold values were, for the most part, found at younger ages; however, there is no clear trend between age and stereo threshold in our dataset. Keep in mind that we specifically recruited nine subjects with stereo vision deficits (these subjects are designated in all figures by solid shapes). This study was not designed to create a normative database.

### Test–Retest Reliability of the Four Stereotests

Figure 2 illustrates the test–retest reliability for the tasks used as scatterplots with dotted lines plotted to represent perfect agreement (on the left) and as Bland–Altman plots (on the right). The test–retest reliability of the ASTEROID test (Figs. 2A, 2B) was quite stable. ASTEROID stereo thresholds correlated very well upon repeated test completions, both between the first two times completing the task (*r* = 0.86, *P* < 0.001, Pearson's correlation on log thresholds) and between the first and third (*r* = 0.68, *P* < 0.001, Pearson's correlation on log thresholds). Bland–Altman analysis showed that the ASTEROID stereo thresholds were very stable, with a mean threshold difference of 0.058 log arcsec (a factor of 1.14) between first and second completions and 0.155 log arcsec (a factor of 1.43) between first and third completions. This shows a slight learning effect; subjects performed better upon follow-up task completions than they did on their first. The 95% limits of agreement from Bland–Altman analysis of ASTEROID stereo thresholds were narrow: ±0.370 log arcsec (a factor of 2.34) between the first two task completions and ±0.580 log arcsec (a factor of 3.80) between the first and third.

The test–retest reliability of the PROPixx standard test (Figs. 2C, 2D) was extremely stable. PROPixx stereo thresholds correlated extremely well upon repeated test completions, both between the first two times completing the task (*r* = 0.90, *P* < 0.001, Pearson's correlation on log thresholds) and between the first and third task completions (*r* = 0.88, *P* < 0.001, Pearson's correlation on log thresholds). Bland–Altman analysis showed that PROPixx stereo thresholds were, as expected, very stable, with a mean threshold difference of 0.002 log arcsec (equivalent to a factor of 1.005) between the first two task completions and 0.108 log arcsec (a factor of 1.28) between the first and third. This indicates, on average, a very slight learning effect between the first time completing the PROPixx tasks and follow-up times completing it; subjects performed slightly better on follow-up task completions than they did on their first. The 95% limits of agreement from Bland–Altman analysis of PROPixx stereo thresholds were quite narrow: ±0.252 log arcsec (a factor of 1.79) between the first two task completions and ±0.300 log arcsec (a factor of 2.00) between the first and third.

The test–retest reliability of the Randot Circles stereotest (Figs. 2E, 2F) might have been influenced by the floor effect of the test. Randot Circles thresholds correlated quite well between the first and second times completing the task (*r* = 0.84, *P* < 0.001, Pearson's correlation on log thresholds), with 20 of 39 subjects scoring the lowest value of 20 arcsec both times. Thresholds between the first and third times completing the Randot Circles task also correlated well, to a lesser degree (*r* = 0.66, *P* < 0.001, Pearson's correlation on log thresholds). Bland–Altman analysis showed that there was minimal variation between Randot Circles thresholds from the first time completing the task and follow-up tasks; the mean threshold difference was –0.006 log arcsec (a factor of 0.986) between first and second task completions and 0.000 log arcsec (a factor of 1.00) between first and third. The 95% limits of agreement from Bland–Altman analysis of Randot Circles stereo thresholds were narrow: ±0.241 log arcsec (a factor of 1.74) between the first two times completing the task and ±0.393 log arcsec (a factor of 2.47) between the first and third.

The test–retest reliability of the Randot Preschool test (Figs. 2G, 2H) was more stable than that of Randot Circles, but its correlation was again probably driven by the floor effect of the test. Randot Preschool thresholds from repeated task completions correlated better than any other stereotest in this study, both between the first two times completing the task (*r* = 0.95, *P* < 0.001, Pearson's correlation on log thresholds) and between the first and third (*r* = 0.82, *P* < 0.001, Pearson's correlation on log thresholds). Bland–Altman analysis showed slightly more variation in the Randot Preschool task than in the Randot Circles task, with a mean threshold difference of –0.003 log arcsec (a factor of 0.99) between first and second times completing the task and 0.047 log arcsec (a factor of 1.11) between the first and third. The 95% limits of agreement from Bland–Altman analysis of Randot Preschool stereo thresholds were narrow as well: ±0.235 log arcsec (a factor of 1.72) between the first two task completions and ±0.370 log arcsec (a factor of 2.34) between the first and third.

### Comparability of Stereo Thresholds Obtained on Different Tests

Figure 3 compares the four stereotests against each other for each subject as scatterplots with lines plotted to represent perfect agreement (on the left) and as Bland–Altman plots (on the right). As described in the Methods section, the geometric means of the three time points of data collection for each subject were used for this analysis. Figures 3A and 3B plot ASTEROID thresholds versus the PROPixx thresholds (the gold standard for this study). There is an excellent correspondence between the two tests (*r* = 0.87, *P* < 0.001, Pearson's correlation on log thresholds), with a systematic upward shift, meaning that subjects obtained slightly smaller thresholds on ASTEROID than they did on the PROPixx standard. A *t*-test confirmed this finding, with a mean difference in log scores (PROPixx – ASTEROID) of 0.085 log arcsec (equivalent to a factor of 1.217); on a paired *t*-test, PROPixx thresholds are slightly but significantly higher (*P* = 0.003). Bland–Altman analysis revealed very good agreement between PROPixx and ASTEROID, with 95% limits of agreement of ±61.9 arcsec.

Figures 3C and 3D plot ASTEROID versus Randot Circles test thresholds. This graph is not linear (*r* = 0.54, *P* < 0.001, Pearson's correlation on log thresholds) because the Randot Circles task had a floor effect at 20 arcsec. Despite the significant correlation between thresholds on the two tests, ASTEROID had an obvious advantage (as shown on the plot) with regard to measuring below Randot's floor. Bland–Altman analysis showed poorer agreement between ASTEROID and Randot Circles; 95% limits of agreement were ±135.1 arcsec.

Figures 3E and 3F plot PROPixx versus Randot Circles test thresholds. This graph is also not linear (*r* = 0.66, *P* < 0.001, Pearson's correlation on log thresholds), again revealing the floor effect of the Randot Circles test at 20 arcsec. Bland–Altman analysis showed moderate agreement between PROPixx and Randot Circles with 95% limits of agreement of ±109.6 arcsec. Of subjects who scored below 100 arcsec on PROPixx, 82.6% obtained the best score, 20 arcsec, on Randot Circles, indicating that the Randot Circles stereotest was not able to capture individual variability in thresholds below approximately 100 arcsec.

Figures 3G and 3H plot PROPixx versus Randot Preschool thresholds. Much like Randot Circles, this graph is not linear (*r* = 0.75, *P* < 0.001, Pearson's correlation on log thresholds) due to the floor effect of the Randot Preschool test at 40 arcsec. Bland–Altman analysis showed 95% limits of agreement of ±166.2 arcsec, suggesting moderate agreement between PROPixx and Randot Preschool.

We have not included plots for ASTEROID versus Randot Preschool, because, given the close agreement between ASTEROID and PROPixx, these are very similar to Figures 3G and 3H. The Pearson's correlation is *r* = 0.64 (*P* < 0.001). The correlation is again limited by the floor effect of Randot Preschool; ASTEROID had the advantage of measuring below Randot Preschool's floor of 40 arcsec. Bland–Altman analysis for ASTEROID and Randot Preschool showed 95% limits of agreement of ±197.1 arcsec.

### Number of Trials Required

**Figure 4.**
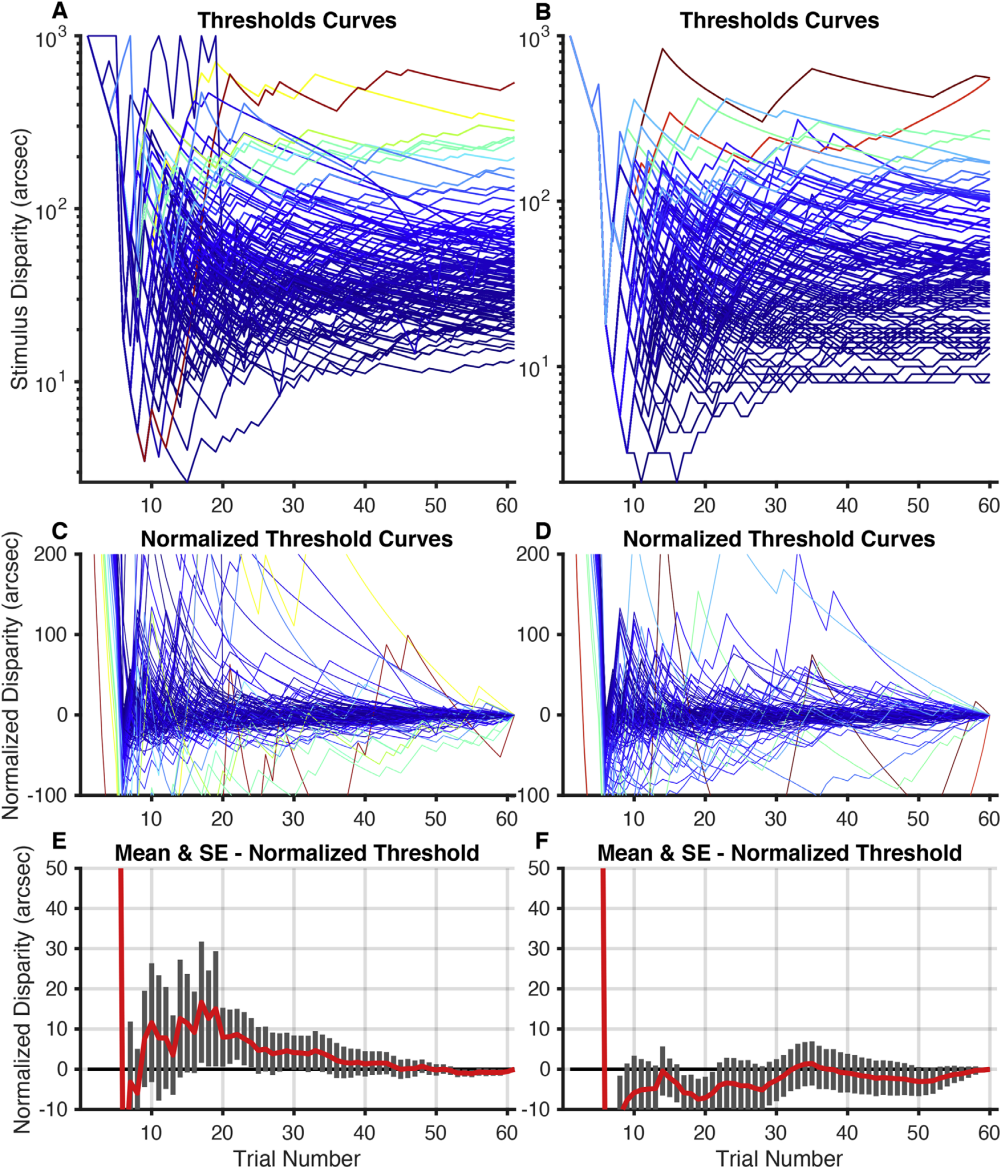
PROPixx and ASTEROID threshold curves. (**A**) PROPixx and (**B**) ASTEROID threshold curves (115), defined as current estimated threshold at each trial, one for each time each subject completed each test. Each *colored line* represents one test completion. (**C**) PROPixx and (**D**) ASTEROID normalized threshold curves (115), normalized to the threshold value determined by the 60th and final trial. (**E**) PROPixx and (**F**) ASTEROID means and standard errors of normalized threshold values for each subject at each trial. The five practice trials with monocular cues are not included in these curves.

PROPixx and ASTEROID both offer more precise stereo thresholds than Randot Circles and Randot Preschool, as neither is restricted to giving one of a set number of possible scores; however, for the 60 trials in this study, both PROPixx and ASTEROID were much more time consuming than these book-based stereotests. This makes it important to determine if all 60 trials are necessary or if reliable stereo thresholds could be obtained with fewer trials. Figure 4 analyzes the PROPixx and ASTEROID (left and right, respectively) threshold curves, defined as the current estimated threshold as a function of the number of trials. Figures 4A and 4B show the threshold curves for 115 completed tasks for each time each subject completed each test (3 × 39 – 2 subjects lost to follow-up after their first visit). Figures 4C and 4D plot the normalized threshold curve (normalized to the 60th and final threshold value) for each time each subject completed each task. If we take the estimate on the 60th trial as the true value, this curve plots the error as a function of the number of trials.

Figures 4E and 4F plot the mean and standard error of the normalized threshold curve graphs in Figures 4C and 4D. One can directly measure from Figures 4E and 4F the amount of average threshold error one would obtain if using a shorter experiment. The standard error bars of the ASTEROID stereotest (Fig. 4F) are very consistent in size throughout the plot, suggesting that the ASTEROID test is very precise no matter the task length.

**Figure 5.**
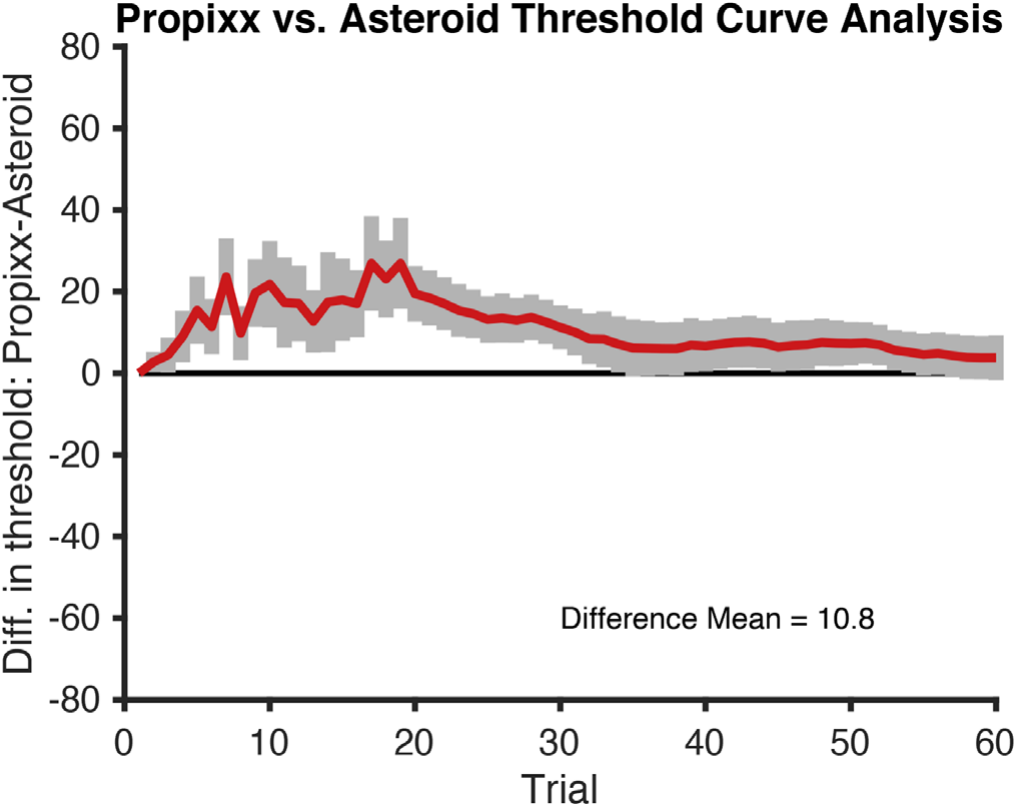
Difference in thresholds, PROPixx versus ASTEROID. Mean difference in thresholds (PROPixx – Asteroid) and standard error bars are plotted for each trial. The five practice trials with monocular cues are not included in this curve.

Figure 5 plots the difference between the PROPixx and ASTEROID stereo threshold estimates as a function of trial number. ASTEROID consistently underestimated the “true” PROPixx threshold, but it is, on average, no more than 25 arcsec lower than the true threshold. From trial 30 onward, the ASTEROID threshold was within 10 arcsec of the PROPixx threshold, determined by using a standard psychophysical method.

### Effect of Viewing Distance Estimate on ASTEROID Results

In this study, we set the viewing distance for ASTEROID to be 45 cm. We relied on the tablet to detect the sticker on the person's forehead and correctly estimate viewing distance. The tablet records the value of this estimate at the end of each trial, and this shows that the estimate was generally accurate, as the mean over trials for each subject was 45.5 cm with a SD of 6.2 cm.

To investigate the effect of these small inaccuracies, we re-ran the ASTEROID staircase on the data for each subject, correcting each trial for a viewing distance of 45 cm. For example, if the tablet estimated the viewing distance as 48 cm and intended to present a disparity of 100 arcsec but the viewing distance was in fact exactly 45 cm, then the disparity actually presented would have been 48/45 × 100 = 107 arcsec. If this persisted throughout the staircase, our corrected threshold estimates would therefore be slightly larger than the original estimate.

This correction did slightly improve the test–retest reliability of ASTEROID, especially across visits. As we saw above, Pearson's correlation coefficient between sessions 1 and 2 (i.e., within the first visit) was 0.86 without correction but became 0.89 after correction. Between sessions 1 and 3 (i.e., between visits), Pearson's correlation coefficient was 0.68 without correction but rose to 0.76 with correction. Similarly, the 95% limits of agreement between sessions 1 and 2 were ±0.37 log_10_ arcsec (a factor of 2.3) without correction and ±0.33 (a factor of 2.1) with correction; between sessions 1 and 3, they were ±0.58 log_10_ arcsec (a factor of 3.8) without correction and ±0.50 (a factor of 3.2) with correction. However, this correction made very little difference to the overall agreement between ASTEROID and PROPixx. The 95% limits of agreement were unchanged to two decimal places, whereas Pearson's correlation coefficient was changed only in the second decimal (0.87 without correction, 0.88 with). Thresholds obtained on ASTEROID were still slightly but significantly lower than on PROPixx (mean difference, 0.082 log_10_ arcsec after correction; *P* = 0.003 on a paired *t*-test). Thus, systematic bias in the estimate of viewing distance by the tablet were not responsible for the slightly lower thresholds observed with ASTEROID.

## Discussion

### Stereotest Comparison

Stereo thresholds determined by the ASTEROID stereotest were very comparable to the PROPixx “true” thresholds. Figure 3A shows that ASTEROID and PROPixx thresholds fall very closely to a 1-1 agreement line, and Figure 3B shows very good agreement between ASTEROID and the PROPixx standard. Figures 3 and 5 show that ASTEROID consistently underestimated the “true” stereo threshold by around 10 arcsec. The reason for this discrepancy is unclear, but, as shown above, it is not because of bias in the estimate of viewing distance by ASTEROID. It may be related to differences in test methodology, including differences in viewing distance (45 cm vs. 120 cm) or display technology, subjects not needing to wear glasses for the ASTEROID stereotest, or subjects needing to physically touch the screen to indicate their answer versus pressing a key on a keyboard for PROPixx (either of which could cause accidental error in answer choice). The fact that the underestimate is particularly noticeable at small thresholds could point to the presentation of sub-pixel disparities as a culprit. At the given viewing distances, the ASTEROID pixels each subtended 52 arcsec whereas each PROPixx pixel subtended 64 arcsec. In both ASTEROID and PROPixx, anti-aliasing was used to present sub-pixel disparities; however, whereas the PROPixx display luminance was linearized, that for ASTEROID was not. Although ASTEROID underestimated subjects’ stereo thresholds, it still compared very closely to a standard measurement of threshold. This suggests not only that ASTEROID is an accurate clinical stereotest but also that it could be used as a less costly, more easily accessible psychophysical stereotest for clinical research.

ASTEROID and Randot Circles are not as comparable (Figs. 3C and 3D); ASTEROID stereo thresholds did not correlate as well with Randot Circles thresholds as they did with those measured by the other two stereotests. This is due in part to the fact that the Randot Circles test assigns thresholds to one of 10 disparity levels with large step sizes between presented disparities, as well as the inability of Randot Circles to measure thresholds below 20 arcsec. Alternatively, ASTEROID reported thresholds as low as 11 arcsec in our study with a much smaller step size that adapted based on subject performance.^20^

Neither Randot Circles nor Randot Preschool correlated as well as ASTEROID with the PROPixx standard (Figs. 3A, 3B, 3E–3H). Both Randot stereotests present disparities with a large step size between them, rather than using smaller step sizes to permit fine discrimination as the PROPixx standard and ASTEROID do. This large step size and lack of fine resolution caused both Randot Circles and Randot Preschool to misrepresent the stereo thresholds of many of our subjects. Neither Randot Circles nor Randot Preschool was administered using a chin rest, but both PROPixx and ASTEROID were; although this testing method was chosen to specifically allow Randot Circles and Randot Preschool to be administered as they typically would be in clinical practice, any differences or modulations in test distance could contribute to the differences in stereo thresholds obtained by these stereotests.

The systematic lower thresholds on Randot Circles compared to the other stereotests (Fig. 1A) may reflect the subjects’ use of monocular circle contour cues to achieve a smaller threshold on Randot Circles than on the other two tests, which do not contain monocular cues. The brain generally optimizes perceptual judgments by combining information from all cues available, which could explain better performance when both stereoscopic and monocular cues are present.^28^ Additionally, differences in stereo threshold as measured by Randot Circles compared to the other stereotests may be due to the specific form of stereopsis that is being targeted; the Randot Circles stereotest probes local stereopsis utilizing some monocular contours, whereas the other stereotests utilize random dot stereograms, either static or dynamic, to probe global stereopsis.

The Randot Preschool threshold curve matches that of the standard PROPixx task much more closely, potentially because it contains no monocular cues and probes global stereopsis and is therefore a more similar task to the PROPixx standard. However, the Randot Preschool stereotest cannot measure thresholds below 40 arcsec; this caused it to incorrectly estimate the thresholds of approximately half of the subjects in our study (Fig. 1A). Additionally, the use of static random dots for the Randot Preschool stereotest as compared to dynamic random dots on PROPixx may have contributed to the difference in threshold estimates.

The Table shows mean thresholds and reliability data for ASTEROID as compared to other current clinical stereotests. The mean threshold for ASTEROID, both in our study and in a previous report of ASTEROID's methods,^20^ is comparable to that of Randot Preschool. The mean threshold of the Randot Circles test is lower than that of the other tests, a finding similar to that of our study.

**Table.** Means and Reliability of ASTEROID, Randot Circles, and Randot Preschool

	Mean Threshold (arcsec)	Population SD (log_10_ arcsec)	Population IQR (log_10_ arcsec)	Bland–Altman 95% Limits of Agreement Between Test and Retest (log_10_ arcsec)	Correlation Between Test and Retest
ASTEROID	66; 57^20^	0.36; 0.35^20^	0.47	±0.37; ±0.64^20^	0.86; 0.63^20^
Randot Circles	32; 29^30^	0.22; 0.15^30^	0.18	±0.24; ±0.34^30^	0.84
Randot Preschool	92; 51^26^	0.35; 0.24^26^	0.00	±0.23; ±0.63^26^	0.95; 0.62^26^

Results are from this and previous studies. Values without reference numbers are from this study. Population statistics use the value on the first measurement; reliability statistics compare the first and second sessions. All values from this paper include all 39 subjects, both stereo-normal and stereo-abnormal. Reference 20 reported Spearman's correlation, whereas this study used Pearson's correlation. Reference 26 reported both Spearman's and Pearson's correlations, but only Pearson's correlation is listed here. This table combines data from studies with different age ranges and inclusion criteria.

### Ideal Number of Trials

For both PROPixx and ASTEROID (Figs. 4E and 4F, respectively), threshold estimates usually did not change substantially beyond around 30 trials. This suggests that time could be saved by using only 30 trials (estimated time of task, 2.5 minutes). If greater accuracy is required, it would be better to have subjects complete several 30-trial staircases and then average the results than to extend the length of a single staircase.

### Test–Retest Reliability

All three clinical stereotests showed stable and similar thresholds among all task completions, with only slight variation between thresholds determined by the first two and first and third task completions. The Bland–Altman limits of agreement for all three stereotests were wider between the first and third task completions than between the first two task completions. This, along with weaker Pearson's *r* correlation coefficients between the first and third completions compared to first two task completions, suggests greater variation between the first and third task completions. This increased variation could be due to subjects performing increasingly better upon repeated testing and therefore learning how to use the testing equipment and how to take each stereotest. It could also be due to subjects being tested on two separate days.

As shown in Figure 2, there was more variation among the ASTEROID stereo thresholds than among thresholds determined by Randot Circles or Randot Preschool; this may have been influenced by floor effects of Randot Circles and Randot Preschool. Because Randot Circles cannot measure any thresholds below 20 arcsec, and in fact measured a threshold of 20 arcsec for 82.6% of subjects who obtained PROPixx thresholds of 100 arcsec or less, many subjects were able to repeatedly obtain a threshold of 20 arcsec each time they completed the Randot Circles stereotest.

Similarly, Randot Preschool does not measure thresholds below 40 arcsec, and subjects were repeatedly able to obtain thresholds of 40 arcsec. Randot Preschool can only distinguish among six levels of stereoacuity; it is better regarded as a binary classifier of normal versus abnormal stereopsis. In this study, with subjects being classified as having normal (60 arcsec or below^29^ as measured by Randot Preschool) or abnormal stereopsis, no subjects changed status at any point.

ASTEROID data, both in our study and in a previous report of ASTEROID methods^20^ (Table), showed the best combination of population interquartile range (larger, capturing the full range of stereoacuity in a normal population) and Bland–Altman limits of agreement (smaller, showing better test–retest reliability). This study obtained better test–retest reliability statistics than the previous report,^20^ possibly because this study used a fixed viewing distance for ASTEROID and because this study computed thresholds after 60 trials rather than 20 as in the previous report. The Randot tests had smaller limits of agreement but also a smaller interquartile range. This is because most subjects with a normal stereo threshold are able to achieve the best threshold value of 20 arcsec on Randot Circles; therefore, there is little discrimination among individuals with different stereo performance. Thresholds on ASTEROID are distributed roughly log normally, in agreement with lab studies of stereoacuity^20^^,^^26^; consistent with this, the interquartile range was approximately 1.35× the standard deviation (Table), as expected for a normal distribution. In contrast, the interquartile range for the Randot tests is less than their standard deviation, reflecting the highly non-normal distribution of results.

It should be noted that our measures of test–retest reliability are free of any inter-examiner differences, as the same examiner measured each subject's stereo threshold all three times it was measured for each stereotest. Because of this, our estimates of test–retest reliability statistics are lower bound; these stereotests may be more variable in clinical practice or research, where a patient may be tested by different examiners at different visits. This applies particularly to Randot Circles and Randot Preschool, which proceed until a subject is no longer willing to give an answer and therefore may be subject to any variability in different examiners’ levels of encouragement during the test. In contrast, ASTEROID uses the same algorithm, full staircase program, and animations every time and should not be as affected by different examiners administering the test.

This study does have a few limitations. Primarily, the number and type of subjects were limited due to our methods of recruiting participants; future studies should focus on recruiting a larger pool of subjects, as well as enough subjects of both normal and abnormal stereoacuity to further investigate the viability and usability of ASTEROID in subjects with various binocular vision anomalies. The methodology of this study was also somewhat limited; the chin rest that was utilized to keep viewing distance stable and standardized among patients would not be helpful for use in clinical practice and may have induced some of the variation in stereo threshold obtained by ASTEROID and PROPixx compared to Randot Circles and Randot Preschool.

## Conclusions

The ASTEROID stereotest is a highly accurate test, correlating more closely to a psychophysical research standard stereotest than other commonly used clinical stereotests. We found that it has good test–retest reliability and can accurately measure the full range of stereo threshold in a normal population, between 1 arcsec and 1200 arcsec.^20^ It has a variety of potential uses. Not only can it quickly (within 30 trials, estimated time of task 2.5 minutes) and easily determine stereo threshold in a clinical setting, but it can also be used in research studies or to track changes during amblyopia or strabismus treatment. ASTEROID offers a simple way to carry out state-of-the-art, laboratory-standard measurements of stereoacuity on a small and portable tablet, which is much less expensive than state-of-the-art, laboratory-standard equipment.
